# Antimicrobial and Antioxidant Potential of *Scenedesmus obliquus* Microalgae in the Context of Integral Biorefinery Concept

**DOI:** 10.3390/molecules27020519

**Published:** 2022-01-14

**Authors:** Maya Margaritova Zaharieva, Dimitrina Zheleva-Dimitrova, Snezhana Rusinova-Videva, Yana Ilieva, Anna Brachkova, Vessela Balabanova, Reneta Gevrenova, Tanya Chan Kim, Mila Kaleva, Almira Georgieva, Milka Mileva, Krassimira Yoncheva, Niko Benbassat, Hristo Najdenski, Alexander Dimitrov Kroumov

**Affiliations:** 1 Department of Infectious Microbiology, The Stephan Angeloff Institute of Microbiology, Bulgarian Academy of Sciences, 26 Acad. G. Bonchev Str., 1113 Sofia, Bulgaria; zaharieva26@yahoo.com (M.M.Z.); illievayana@gmail.com (Y.I.); tanya_85@abv.bg (T.C.K.); milakalevavet@abv.bg (M.K.); 2 Department of Pharmacognosy and Pharmaceutical Botany, Faculty of Pharmacy, Medical University of Sofia, 2 Dunav Str., 1000 Sofia, Bulgaria; dzheleva@pharmfac.mu-sofia.bg (D.Z.-D.); vbalabanova@pharmfac.mu-sofia.bg (V.B.); rgevrenova@pharmfac.mu-sofia.bg (R.G.); 3 Laboratory Cellular Biosystems, Department of Biotechnology, The Stephan Angeloff Institute of Microbiology, Bulgarian Academy of Sciences, 139 Ruski Blvd., 4000 Plovdiv, Bulgaria; jrusinova@abv.bg; 4 Laboratory of Bio-Conversion and Biosynthesis of Microbial Metabolites, Department of Biotechnology, The Stephan Angeloff Institute of Microbiology, Bulgarian Academy of Sciences, 26 Acad. G. Bonchev Str., 1113 Sofia, Bulgaria; abratchkova@yahoo.com; 5 Department of Virology, The Stephan Angeloff Institute of Microbiology, Bulgarian Academy of Sciences, 26 Acad. G. Bonchev Str., 1113 Sofia, Bulgaria; almirageorgieva@gmail.com (A.G.); milkamileva@gmail.com (M.M.); 6 Institute of Neurobiology, Bulgarian Academy of Sciences, Bl. 23 Acad. G. Bonchev Str., 1113 Sofia, Bulgaria; 7 Faculty of Pharmacy, Medical University of Sofia, 2 Dunav Str., 1000 Sofia, Bulgaria; krassi.yoncheva@gmail.com (K.Y.); nbenbassat@pharmfac.mu-sofia.bg (N.B.)

**Keywords:** photobioreactors, modelling, integral biorefinery concept, *Scenedesmus obliquus*, dicholormethane extracts, food-borne pathogens, drug combinations, oregano oil, antioxidant capacity, in vitro cytotoxicity

## Abstract

Small-scale photobioreactors (PBRs) in the inoculum stage were designed with internal (red or green) and external white LED light as an initial step of a larger-scale installation aimed at fulfilling the integral biorefinery concept for maximum utilization of microalgal biomass in a multifunctional laboratory. The specific growth rate of *Scenedesmus obliquus* (Turpin) Kützing biomass for given cultural conditions was analyzed by using MAPLE software. For the determination of total polyphenols, flavonoids, chlorophyll “a” and “b”, carotenoids and lipids, UHPLC-HRMS, ISO-20776/1, ISO-10993-5 and CUPRAC tests were carried out. Under red light growing, a higher content of polyphenols was found, while the green light favoured the flavonoid accumulation in the biomass. Chlorophylls, carotenoids and lipids were in the same order of magnitude in both samples. The dichloromethane extracts obtained from the biomass of each PBR synergistically potentiated at low concentrations (0.01–0.05 mg/mL) the antibacterial activity of penicillin, fluoroquinolones or oregano essential oil against the selected food-borne pathogens (*Staphylococcus aureus*, *Escherichia coli* and *Salmonella typhimurium*) without showing any in vitro cytotoxicity. Both extracts exhibited good cupric ion-reducing antioxidant capacity at concentrations above 0.042–0.08 mg/mL. The UHPLC-HRMS analysis revealed that both extracts contained long chain fatty acids and carotenoids thus explaining their antibacterial and antioxidant potential. The applied engineering approach showed a great potential to modify microalgae metabolism for the synthesis of target compounds by *S. obliquus* with capacity for the development of health-promoting nutraceuticals for poultry farming.

## 1. Introduction

The biorefinery concept is not new. The growing human population and industrial consumption of resources forced the creation of new concepts in biotechnology, “green” and “white biorefinery concept” [1,2,3,4,5]. The microalgae biorefinery concept involves an effective hybrid methodology [6] because the use of biomass from algae as a single technology is a very expensive approach. The application of this concept in every step of the microalgae process development and unit operations by using the principle of analogy and minimizing scientific efforts and costs and finding the cheapest substrates is published in our previous works [7,8,9] where system analysis theory and modelling approach [10,11,12] guided us. Hence, the integral use of all microalgae products such as proteins, lipids, carbohydrates and high-value products (HVP) (e.g., carotenoids, astaxanthin, antimicrobials, antivirals and antifungals products, etc.) will allow the realization of market competitive microalgae technology. Our experience in secondary metabolite production [13] helped us to plan the experiments in the current study.

After the creation of innovative PBRs in the incubation production stage, the key problem to solve was to select the algae strains with the capacity to synthesize multiple products and thus to fulfil the concept of algae biorefineries. Our choice of interest in this study was the still unstudied *Scenedesmus obliquus* strain 8610 isolated in Bulgaria and preserved in the National Algae Culture Collection-Plovdiv (PACC). The state-of-the-art *S. obliquus* showed promising potential in synthesizing biologically active compounds [14,15,16,17,18,19]. Hence, identification of BAC (biologically active compounds) with many activities obtained from *S. obliquus* strains via the investigation of extracts from microalgae biomass remains challenging. More details are discussed below in the current state of the art for this complex system.

The application of various microalgal species in the form of dried biomass or extracts and bioactive compounds thereof has been applied for a long time in animal husbandry, and especially in poultry farming, to enhance animal health and performance, due to their nutritional properties and immune-stimulatory, antioxidant and antibacterial activities [20,21,22,23,24,25,26]. The most commonly used microalgal species for this purpose are different *Chlorella* [20,22,24] and *Spirulina* [20,21,23] spp. In the light of the growing urgent need to limit the use of antibiotics as health-promoting agents because of the development of bacterial resistance, it is even more necessary to search for new opportunities to replace existing practices with healthier diets.

In general, there are few reports in the scientific literature on the use of *Scenedesmus* spp. In poultry farming as a dietary supplement with antibacterial properties in the diet of broilers, [25,27,28], and as far as we know, there are no data about the application of *Scenedesmus obliquus* extracts for such purposes. *Scenedesmus* spp. extracts generally have varying antibacterial activity, and the effect is higher against Gram-positive bacteria. There are several studies dedicated to the antimicrobial effect of *S. obliquus*, and some of them include a few of the most relevant food-borne pathogens such as *Escherichia coli*, *Staphylococcus aureus*, *Pseudomonas aeruginosa* and *Salmonella* sp. Regarding the agar diffusion method, the best results have been achieved against *S. aureus* (inhibition zone 26 mm) and *E. coli* (inhibition zone 23 mm) with methanol extracts (0.1 mg) when the concentration of phosphorus (which is the most nutritional factor that affects the metabolism and cell growth) was 0.01 and 0.007 g/L, respectively [29]. The diethyl ether crude extract recorded 19.5 and 18.5 mm inhibition zones against *S. typhi* and *P. aeruginosa*, respectively, and 12.5–19.5 mm inhibition zones and minimal inhibitory concentrations MIC 0.5–1.2 mg/mL against *Bacillus cereus*, *S. aureus*, *E coli* and *Klebsiella pneumoniae*. That extract had inhibition zones of 18.3 and 15.7 mm against the mycotoxigenic fungi *Aspergillus steynii* and *A. carbonarius*, respectively, and the zones against *A. ochraceus*, *Fusarium verticillioides*, *Penicillium verrucosum*, *A. flavus*, *A. parasiticus*, *A. westerdijikia* and *F. proliferatum* were smaller but over 8.7 mm. The aqueous, chloroform, ethyl acetate and hexane extracts had weaker antimicrobial effects [30]. An ethyl acetate extract had an inhibition zone of 11 mm against *S. aureus* and 9 mm against *E. coli*, while the ethanol extract was not active [31]. Lipids and fatty acids but not the culture medium from the microalga inhibited *S. aureus* and *Streptococcus pyogenes* with inhibition zones of 16 mm and 18 mm, respectively. No activity towards *P. aeruginosa, B. cereus*, *E. coli*, *Yersinia enterocolitica*, *Salmonella typhimurium* and *Candida albicans* was observed [32]. In other studies there was also no activity of the methanol extract, the pellet itself, or the extracellular supernatant against *B. subtilis*, *E. coli*, *P. fluorescens*, *C. albicans* or *Saccharomyces cerevisiae* [33], nor did the ethanol, methanol and hexane extracts from the biomass and supernatant display any activity towards *Vibrio fisheri*, *Aeromonas fluvialis*, *B. subtilis* subsp*. subtilis*, *Enterococcus faecalis*, *E. coli*, *P. aeruginosa*, *Sphaerotilus montanus*, *Spirillum winogradskyi*, *S. aureus* subsp*. aureus* and the fungi *A. niger* and *Wallemia sebi* [34]. The photometric method recorded pronounced antagonistic activity of an aqueous extract against four test strains of opportunistic bacteria—*E. coli, K. ozaenae* (50% growth inhibition)*, P. aeruginosa* (>82% growth inhibition) and *S. aureus* [35]. A visual assessment for turbidity of mixtures of pathogens and the microalgae in a medium showed that intracellular (food-grade solvent) and extracellular ethanol–water (1:1) extracts exerted low or mild inhibition against *Salmonella* sp., *S. aureus*, *P. aeruginosa* and *E. coli*, and two strains from *S. obliquus* had different effects [36]. The contradictory results obtained in these studies indicate the need for more research on the antibacterial activity of different extracts from S*. obliquus* biomass by using different solvents to maintain consistency with published data.

The favourable environment in which microalgae live and develop is characterized by constant exposure to light and oxygen, so the adaptation processes are an excellent prerequisite for the over-generation of ROS, as well as some other oxidative-active structures. Normally, cellular metabolism in living organisms naturally produces reactive oxygen species (ROS) in respiratory processes. ROS are first and foremost described by many authors as potentially harmful agents. They are unstable molecules, and their over-generation causes reactions with other molecules in a cell followed by harmful effects. The process of mismanagement of ROS cascade has been associated with the progression of many serious pathological conditions, including ischemic events [37], cancer [38], etc. It has been demonstrated that cancer cells contain ROS/oxidative stress-mediated defects in the mtDNA repair system and histone protection [38]. The biological effects of ROS in aerobic organisms are controlled by a range of physiological antioxidant defence mechanisms, which involve a complex group of processes, all aimed at preventing or retarding excess oxidation at the cellular level. Antioxidants are ROS scavengers that can shield, scavenge and repair oxidative damage, thereby defending target assemblies or molecules from oxidative damages. According to the mode of action, antioxidant protection in the body can be realized in (i) an enzymatic way or (ii) through primary or (iii) secondary antioxidants. Antioxidant enzymes are superoxide dismutase, glutathione peroxidase, glutathione reductase and catalase. Primary antioxidants cancel free radicals by two mechanisms. One is through the donation of an H-atom, known as the hydrogen atom transfer (HAT), and the other is through single-electron transfer (SET) mechanisms [39].

The adaptation of algae to environmental conditions presupposes the presence of a stable system of antioxidant defence. In fact, many studies have shown that algae are rich in protective enzymes and antioxidant molecules, including tannins, ascorbic acid, tocopherols, carotenoids, phospholipids, chlorophyll-related compounds, bromophenols, catechins, polysaccharides, etc. There are data showing the good ability of seaweed extracts to inhibit lipid peroxidation, to scavenge free radicals, and/or to prevent their generation [40,41,42]. They are also rich in polyunsaturated fatty acids, which are an excellent target for oxidative processes [43,44,45]. The fact of their stability to oxidation during storage and processing is also indisputable, which means that they have serious protective antioxidant systems in their cells. Microalgae can accumulate vitamin E, fat-soluble compounds and polyphenols with antioxidant properties [46]. Microalgal biomass is considered a multicomponent antioxidant system that is part of the antioxidant protection strategy and generally more effective than the pure compounds due to the resulting interactions between different antioxidant components [47]. Based on the scientific data cited above, we set for ourselves the goal to investigate the antibacterial activity along with the antioxidant capacity of extracts obtained from the *S. obliquus* strain 8610 (National Algae Culture Collection, Plovdiv, Bulgaria) chosen for investigation during cultivation stages by using flexible innovative approaches. An important part of that goal was to check the ability of maximum utilization of the microalgal biomass starting from the initial steps of the cultivation process in the frame of the biorefinery concept. Usually, high light intensity is used in combination with other stress factors. Such strategies include a two-stage cultivation system, as well. Many research papers and reviews are available that show high light intensity as a main stress factor for the synthesis of secondary metabolites [48,49,50]. This is valid and has a techno-economical meaning in a bigger scale of production stages but not in the incubation stage. Regarding the latter stage, for the culturing of *Scenedesmus* spp., there are many works confirming that high as well as low irradiance supports secondary metabolite synthesis [51], which makes the search space for the optimal light conditions wider. In order to avoid expensive and numerous experiments when researching the unstudied *Scenedesmus obliquus* strain, we focused on the achievements of published works and our previous experience with low external LED white light intensities. Therefore, we applied a combination of LED external white and internal green or red lights. The effectiveness of the cultivation approaches was evaluated by the determination of total polyphenols, flavonoids, chlorophyll a and b, carotenoids and lipids. The solvent dichloromethane was used for the extraction of non-polar compounds, keeping in mind that long-chain free fatty acids are some of the most potent antibacterial compounds isolated from microalgae, and they are also frequently used in most animal diets [26,31,52,53,54]. The antibacterial and antioxidant potential of the dichloromethane extracts obtained from the *S. obliquus* biomass cultured under the combination of internal (green and red) and external white LED light was investigated in parallel. Three food-borne pathogenic bacterial species (*S. aureus, E. coli* and *S. typhimurium*), which are known to cause infections in broilers and the contamination of meat and meat products with a subsequent significant economic burden for the poultry industry, were selected as target microorganisms [55,56,57,58]. As far as microalgae are widely used as nutraceuticals and thus co-administered with other dietary supplements or drugs, the extracts were combined with clinically approved antibiotics/chemotherapeutics or oregano essential oil (OrO) in order to evaluate the combination effects and their potential use for prevention and treatment of infectious diseases as ingredients of food supplements or in therapeutics schemas. The selected antibacterial drugs belong to two different pharmacological groups of antibiotics or chemotherapeutics (penicillins and fluoroquinolones) that are commonly used in poultry farming to control bacterial infections [44,59,60] and included penicillin, ciprofloxacin and enrofloxacin. OrO is well known for its strong antibacterial activity against a large number of Gram-positive and Gram-negative pathogenic bacterial species, including numerous food-borne pathogens [61,62,63,64], which is why it is widely used in the food industry [61,65,66,67,68,69]. There are also several studies proving its beneficial effects on the body weight and feed conversion ratio, intestinal health and the antioxidant status in broilers [70,71,72] and growing ducks [73]. It should be noted that OrO is also a very strong antioxidant due to the presence of carvacrol and thymol [74,75]. Therefore, we assumed that a synergistic combination between microalgal extracts and OrO can represent a promising option for the replacement of antibiotics in the poultry industry as a growth promoter with antibacterial and antioxidant potential.

## 2. Results

### 2.1. Growth Rate and Phytochemical Analysis of S. obliquus Biomass Grown in Innovative Small-Scale PBRs under Internal (Red or Green) and External White LED Light

**Figure 1:**
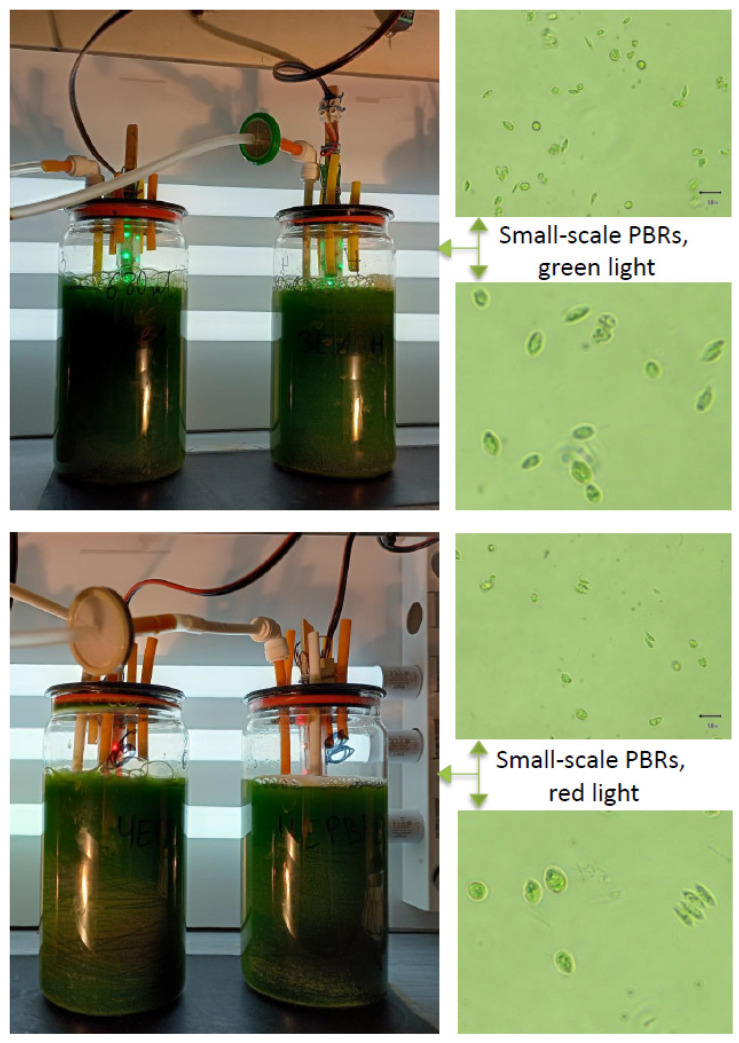
Small-scale PBRs with internal light used in this study.

The experiments with the *S. obliquus* strain were performed in small-scale PBRs (SC-PBRs) under internal green (SC-PBR1) and red (SC-PBR2) light illumination with external irradiation by white LED light behind each reactor in order to initiate intensive synthesis of biologically active compounds in the microalgae cells (Figure 1). The culturing period lasted 23 days until the culture reached the stationary phase. The morphology of cultures was monitored visually under an inverted biological microscope (BOECO Model BIB-100, Hamburg, Germany), magnification 200 and 400*×*.

The experiment in the SC-PBR1 supplied with internal green light was started with an initial biomass concentration of *X*(0) = 0.32 g/L for corresponding time *t*(0) = 0. The second experiment in the SC-PBR2 supplied with internal red light was started with an initial biomass concentration of *X*(0) = 0.41 g/L for corresponding time *t*(0) = 0. Operational conditions were in intervals as follows: room temperature *T*_room_ = 20–28 °C; temperature inside the SC-PBRs = 24–28 °C; pH = 7.4–10; Q_CO2_ = 2–10% [*v*/*v*]; air flow enriched with CO_2_ *Q*_air_ = 0.1–0.4 L/L/min; nutrient medium was Kroumov’s medium composition [76] of the modified M-8. At the end of the logarithmic growth curve (day 29) the culture in the SC-PBR1 reached a biomass concentration of 6.95 g/L, whereas the culture in SC-PBR2 reached a biomass concentration of 5.87 g/L (see Table 1).

The specific growth rate (SGR) in the exponential phase was calculated according to Equation (1):

(1) $$\mu =\frac{\left(\ln\left(X(2\right)\right)-\ln\left(X(1\right)))}{\left(t\left(2\right)-t\left(1\right)\right)};\left[\frac{1}{d}\right]$$

- For SC-PBR1:

*μ*(SC-PBR1)= 0.039 *d*^−1^ -**>** SGR (specific growth rate during logarithmic growth)

*X*(2)= 6.95 [g/L]; *t*(2) = 29; *X*(1) = 2.85 [g/L]; *t*(1)= 6 [*d*]; time interval 23 days;

where

*X*(2) = 6.95 [g/L] stands for the second point of biomass concentration from the logarithmic growth curve with corresponding time *t*(2)= 29 [*d*];

*X*(1) = 2.85 [g/L] stands for the first point of biomass concentration from the logarithmic growth curve with corresponding time *t*(1) = 6 [*d*].

- For SC-PBR2:

*μ*(SC-PBR2) = 0.043 *d*^−1^ -> SGR (specific growth rate during logarithmic growth)

*X*(2) = 5.87 [g/L]; *t*(2) = 29 [*d*]; *X*(1) = 2.18 [g/L]; *t*(1) = 6 [*d*]; time interval 23 days;

where

*X*(2) = 5.87 [g/L] stands for the second point of biomass concentration from the logarithmic growth curve with corresponding time *t*(2) = 29 [*d*];

*X*(1) = 2.18 [g/L] stands for the first point of biomass concentration from the logarithmic growth curve with corresponding time *t*(1) = 6 [*d*].

Note: the biomass concentrations *X*(0) = 0.32 and *X*(0) = 0.41 stand for the inoculum in SC-PBR1 and SC-PBR2, respectively, with corresponding *t*(0), which stands for beginning (start) of the cultivation process in both reactors. Zero (0) points are not included in the calculation of SGR (see Equation (1)).

SGR (*µ*) in the above Equation (1) is a solution of differential Equation (2) (see Materials and Methods, Section 4.2) where the key assumption is that *µ* = const at the logarithmic growth phase of the growth curve for the given period of time and operational conditions. This assumption means that state parameters such as T, pH, light irradiation and macro- and micronutrients of the medium do not limit the growth during culturing cells in the logarithmic phase of the growth curve [8]. Hence, under the above assumption, Equation (2) can be solved and a form of Equation (1) can be obtained. It should be noted that from day 0 up to day 6 of the growth curve, the SGR is high, and this represents an acceleration phase after the Lag phase. During this period the biomass concentration is very low, and the light penetrates in a radial direction from a wall to the other wall of the PBR column. Analysing the growth curve from the 29th to the 36th day of the cultivation period after the exponential phase (see Table 1), we observed a slight decrease in the biomass concentration in both PBRs, which is evidence that the cells entered stationary phase under these conditions and PBR design. The macro- and microelements of the nutrient medium had most likely been utilized by the cells, and a limitation by one of them had occurred. On the other hand, light irradiation also can be a limiting factor in the photoautotrophic growth because at such high biomass concentrations (between 5 and 7 g/L) light penetration depends on the Beer–Lambert law and decreases in direction from the wall to the centre of the PBRs [8].

The combination green(internal):white(external) promoted more accumulation of flavonoids in *S. obliquus* cultivated in SC-PBR1 (Table 2). Polyphenol content was higher under the combination red(internal):white(external) in SC-PBR2. However, although statistically detectable, the differences between both PBRs are not biologically significant. Except for chlorophyll b, no impact on the pigments production was observed. The light irradiation conditions in this experiment did not affect the content of lipids and carotenoids in the biomass in either PBRs.

As a next step, two dichloromethane extracts were obtained from the biomass collected from each SC-PBR—one (E1) from biomass cultured under green(internal) and white(external) light and a second (E2) from biomass cultured under red(internal) and white(external) light. Both extracts were screened for fatty acids and carotenoids (Figures S1–S8). The strategy for compound recognition was based on the fragmentation patterns and diagnostic ions for carotenoids and fatty acids, compared with literature data [77,78,79]. The main points in the peak’s annotation and dereplication are: (1) accurate masses in Full MS and ddMS2, (2) MS/MS fragmentation patterns, (3) relative abundance of precursor and fragment ions, (4) elemental composition, (5) consistency with the simulated monoisotopic profiles, and (6) comparison with the fragment spectra and chromatographic behaviour of literature data. The monoisotopic profile in the Full MS spectrum and MS/MS spectra of some of the annotated compounds are depicted in the supplemental material (Figures S1–S8).

The MS/MS spectrum of **1** with a protonated molecule [M + H]^+^ at *m/z* 537.436 was acquired (Table 3) (Figure S1). The precursor ion yielded an abundant ion at *m/z* 114.0914 [(M + H) − C_32_H_39_]^+^ (38.6%) together with fragment ions at *m/z* 406.341 [(M + H) − C_10_H_11_]^+^, 198.185 [(M + H) − C_26_H_27_]^+^ and 69.071 [(M + H) − C_10_H_11_]^+^. In (−) ESI-MS/MS, **1** gave an adduct at *m/z* 581.429 [M + HCO_2_H]^−^ and [M − H]^−^ at *m/z* 535.424 (Figure S1). The fragmentation pattern of **1** was consistent with carotene isomers. Compound **3** afforded [M + H]^+^ at *m/z* 567.418 and a fragment at *m/z* 549.407 formed by the elimination of water from the protonated molecule. In addition, abundant fragment ions were generated at *m/z* 145.101 [(M + H) − C_29_H_42_O_2_]^+^, 119.086 [(M + H) − C_31_H_44_O_2_]^+^ and 93.070 [(M + H) − C_33_H_46_O_2_]^+^ (Figure S2). An ion at *m/z* 147.116 was observed, as was previously observed in ketocarotenoids containing a hydroxyl group C-3 and a keto group in C-4 in the β-ring [77]. Thus, **3** was putatively identified as hydroxyechineone (Figure S2). In Full MS spectra, [M + H]^+^ at *m/z* 565.404 was recorded, which is in accordance with canthaxanthin (Figure S3). [M + H]^+^ at *m/z* 569.4353 could be assigned to isobars lutein/zeaxanthin. Two peaks with [M + H]^+^ at *m/z* 601.4251 and 601.4208 accompanied with [M]^+^ at *m/z* 600.4159 and 600.4156 were ascribed as neoxanthin/violaxanthin [77].

In negative ion mode, one polyunsaturated fatty acid (PUFA), five monohydroxy, five dihydroxy, and three trihydroxy-PUFAs were tentatively identified in both extracts (Table 2). Compound **10**, with deprotonated molecule at *m/z* 275.201 (C_18_H_28_O_2_), gave a diagnostic ion at *m/z* 231.211 ([M − H − CO_2_]^−^, indicative of the presence of a carboxylic group. Based on accurate mass, molecular formula and the fragmentation pathway, **10** was tentatively related to octadecatetraenoic acid (stearidonic acid), previously found in *Spirulina* [78] (Figure S4). The isobaric pair **12**/**13** shared the same [M − H]^−^ at *m/z* 293.212 and revealed fragment ions at *m/z* 275.201 [M − H-H_2_O]^−^ and 231.211 [M − H-H_2_O − CO_2_]^−^. Thus, **12**/**13** were tentatively identified as hydroxyoctadecatrienoic acid and its isomer [79] (Figure S5). In the fragmentation pathway of **14**, the consequent neutral losses at *m/z* 287.165 [M – H − H_2_O]^−^ and 269.155 [M − H − 2H_2_O]^−^ suggested the presence of two hydroxyl groups, and **14** could be ascribed to dihydroxyoctadecapentaenoic acid [79] (Figure S6). Concerning **18**, the fragment ions at *m/z* 305.176 [M − H − H_2_O]^−^, 287.165 [M − H − 2H_2_O]^−^ and 95.0487 [C_6_H_9_O_2_ − H_2_O]^−^ were indicative of the presence of three hydroxyl groups in the structure. The fragmentation pathway revealed a subsequent loss of CH_2_-groups. Thus, **18** could be related to trihydroxyoctadecatetraenoic acid (Table 2) [79] (Figure S7). In the same manner, five hexadecanoic PUFAs (**6**, **7**, **8**, **10**, **11**) were tentatively identified (Table 2) (Figure S8).

### 2.2. Antimicrobial Activity of S. obliquus Extracts

The antibacterial activity of both extracts was tested on the following three food-borne pathogens: *S. aureus, E. coli* and *S. typhimurium*. The results from the BMD test showed that the minimal inhibitory concentration of both extracts was 12.5 mg/mL for *E. coli* and *S. typhimurium* or higher for *S. aureus*. The metabolic (redox, dehydrogenase) activity of treated bacteria was measured for both extracts. It was significantly lower for E2 (1.09–5.2%) than E1 (10.4–16%) as presented in Table 4. The working solutions of the extracts were at the concentration of 200 mg/mL (in 96% ethanol), whereby the final concentration of ethanol for the highest applied concentration of the extract (12.5 mg/mL) after dilution with the bacterial suspension was 6%. Ethanol does not inhibit bacterial growth in concentrations up to 25%, as published previously by our group [80], and therefore no additional control with 6% ethanol was used.

### 2.3. Combination Effects between Dichloromethane Extracts of S. obliquus Biomass and Penicillin, Fluoroquinolones or Oregano Essential Oil

Each of the two extracts was combined with penicillin (PEN), fluoroquinolones (ciprofloxacin—CIP, or enrofloxacin—ENR) or OrO. First the single effects of PEN, CIP, ENR and OrO were determined (Table 5). The MICs of PEN, CIP and ENR correlated to the EUCAST data about the sensitivity of the tested bacterial strains [81]. The MIC of OrO for *S. typhimurium* was determined to be 0.05%. The MICs for *S. aureus* and *E. coli* were confirmed as published before [64].

The results from the combinations are presented in Table 6, according to the recommendations for the calculation of results obtained from the checkerboard assay [82,83]. The effective MICs of the extracts in the combinations (MIC_C_), which diminished the MICs of PEN, CIP, ENR and OrO two- (additive effect) or four-fold (synergism) ranged between 0.005 and 0.025 mg/mL. Only the combination between each of the microalgal extracts and ENR on *S. aureus* did not potentiate the antibacterial effect of the chemotherapeutic and the calculated effect was found to be “indifference”. The DEHA activity of the synergistic and additive combinations ranged between 0.3 and 36% (data not shown in the table), which suggests a bacteriostatic effect.

### 2.4. Antioxidant Capacity

The cupric-reducing antioxidant capacity (CUPRAC) of both extracts was calculated according to Apak et al. [84] and presented as mmol Trolox equivalents/g dry extract (TE/g) as follows:

*E*1 = 0.0391 ± 0.0020 mmol TE/g *E*2 = 0.1385 ± 0.0024 mmol TE/g

**Figure 2:**
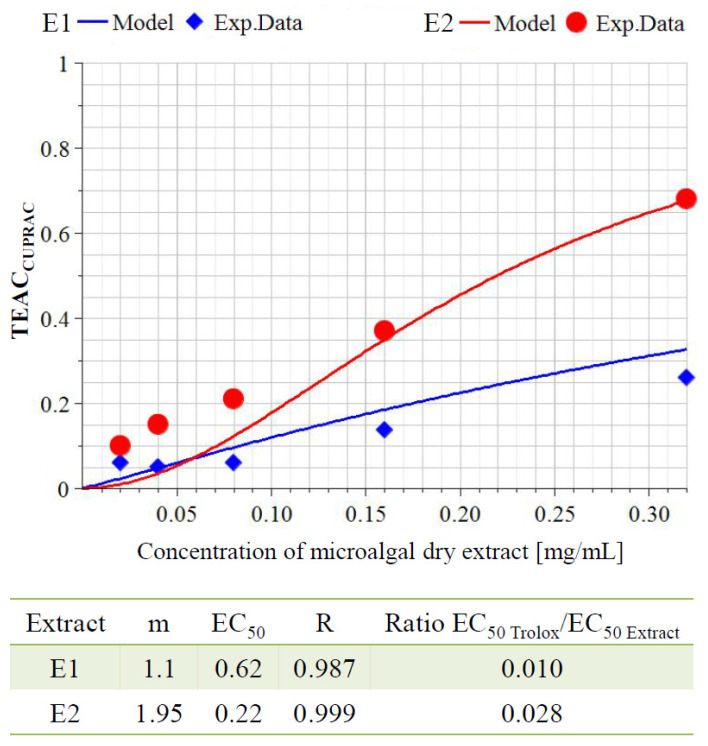
Cupric ion-reducing antioxidant capacity (CUPRAC) assay of *E*1 and *E*2. The ability of extracts to reduce cupric ions (CUPRAC) is expressed as mmol Trolox equivalent antioxidant capacity (TEAC_CUPRAC_) and is plotted on the *Y*-axis. Legend: EC_50_—median effective dose of the microalgal extracts achieving 50% cupric-reducing antioxidant capacity compared with the reference substance Trolox; *m*—hillslope; *R*—coefficient of correlation.

In addition, the ability of both extracts to act as reducers of Cu (II) ions is presented graphically in Figure 2. Trolox was used as a reference substance, the maximum activity of which was assumed to be 1 (corresponding to 0.075 mM or 0.02 mg/mL Trolox), and the activities of the test samples were calculated as mM Trolox equivalent antioxidant capacity (TEAC_CUPRAC_). Both of the tested extracts showed a well-defined concentration–effect relationship in the concentration range of 0.05–0.3 mg/mL dry extract. The extract E2, obtained from the biomass richer in polyphenols, showed a higher activity as a reducer of copper ions (EC_50_ = 0.22 mg/mL). The correlation coefficient *R* for both extracts *E*1 and E2 was above 0.98, which represented an excellent fit of the experimental data and proof of the very exact calculation of the EC_50_ value. The EC_50_ value of the Trolox was 0.025 mM (0.00625 mg/mL). The ratio between the EC_50_ of Trolox [mg/mL] and *E*2 [mg/mL] was almost three-fold higher (0.028) than that (0.01) between the EC_50_ [mg/mL] of Trolox and E1 [mg/mL] [85].

### 2.5. In Vitro Cytotoxicity of S. obliquus Dichloromethane Extracts

**Figure 3:**
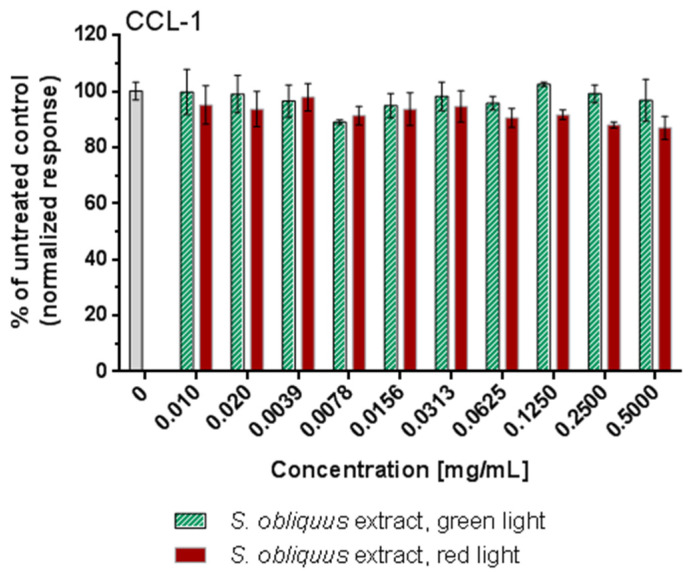
In vitro cytotoxicity of *S. obliquus* extracts obtained from biomass cultured under green or red light.

The in vitro cytotoxicity of E1 and E2 was tested on the CCL-1 cell line (normal mice fibroblasts) for an incubation period of 72 h. The tested concentrations were in the range 0.01–0.5 mg/mL. As presented in Figure 3, all concentrations were not toxic for the cells as far as the cell survival fraction varied from 86% to 100% in a concentration-dependent manner. There was no significant difference between the untreated control and the treated groups except for 0.25 and 0.5 mg/mL *E*2 (*p* < 0.005). However, these two concentrations are still not cytotoxic according to ISO 10993-5/2009 [86]. Morphological evaluation of the treated samples revealed no signs of cytotoxicity.

## 3. Discussion

This work highlighted the engineering approach of putting complex studies into practice to fulfil the integral biorefinery concept. The upstream operations in the inoculum stage allow the confirmation of the hypothesis of whether the chosen strain *S. obliquus* grows under different nutrients medium in an innovative small PBR type with a supply of different combinations of external and internal light irradiation. In this stage, the tolerance of the strain to the elevated CO_2_ content can also be checked when the goal is the maximization of secondary metabolites synthesis. The creation of a multifunctional laboratory allows us to quickly check different hypotheses about the overproduction of BAC in parallel experiments related to analysis of biomass and extracts obtained from the target *S. obliquus* strain in many directions. In the state-of-the-art development, the uninvestigated strain passed through many phases of research in incubation and production stages.

It must be noted that a remarkably high biomass concentration in both SC-PBR1 and 2 in the incubation stage was achieved. The supply of the SC-PBRs with air flow without an additional supply of CO_2_ or with a very low concentration of CO_2_ that do not correspond to the substrate utilization needs for different biomass concentrations results in the lowering of the SGR. For example, when *X* < < 0.5 g/L (the biomass concentration is very low and the culture starts growing in logarithmic phase), then a supply of 2–10% is not effective because its utilization corresponds to the simple relationship *X*(*t*) − *X*(*t* − 1) = *Y*_co2/x_(CO_2_(*t*) − CO_2_(*t* − 1)), where *t*-stands for the time [*d*] in the logarithmic growth phase; *Y*_co2/x_ is the yield coefficient showing how much CO_2_ is consumed to form 1 g biomass *X*. Hence, when the difference of *X*(*t*) − *X*(*t* − 1) in the biomass increase is very low at the beginning of the cultivation in the logarithmic growth phase (for example, *X*(1) = 0.05 g/L and *X*(2) = 0.1 g/L for the given time of the logarithmic growth phase, *t*(2) = 2 days stands for the second point in the logarithmic growth phase and *t*(1) = 0 stands for beginning of the cell growth in logarithmic phase); then the consumption of CO_2_ is very low and does not require a high supply such as 2% up to 10% CO_2_. If *X*(4) − *X*(3) is high (for example, *X*(4) = 5 g/L and *X*(3) = 2 g/L for the given time *t*(4) − *t*(3)) in the logarithmic growth phase, then CO_2_ supply has to be controlled accordingly between 2% and up to 10% CO_2_. Note: any point where samples are taken for the determination of biomass concentration corresponds to the cell growth in the logarithmic growth phase. A very good indicator of CO_2_ access in the liquid is the pH value. An increasing value above the set point pH value means CO_2_ is a limiting factor. Additionally, vice versa, a decreasing pH value means CO_2_ supply is higher than its consumption. Hence, pH value can be used as a control strategy for CO_2_ supply. On the other hand, the problem was not the uniform distribution of liquid flow and low mixing speed, which allowed sedimentation of a part of the biomass and lowering of the SGR, as well. As might be expected, the goals of the inoculum stage do not claim to give optimal conditions for obtaining high-density culture. Hence, the key parameters for BAC were recognized to be light irradiation and the application of external and internal sources of light because CO_2_ concentration and the nutrient medium (in this case) are not considered to be a stressful factor of secondary metabolite biosynthesis. In this stage, as mentioned, only hypotheses about BAC synthesis are checked through parallel sets of cell cultivation—applying eight small innovative PBRs with internal blue, green, red and white light, aiming to minimize research efforts and money. For example, experiments with internal green (SC-PBR1) and red light (SC-PBR2) checked the potential of unstudied *S. obliquus* to synthesize secondary metabolites. The experiments with extracts obtained from biomass grown in combinations of white(external):green(internal) or white(external):red(internal) conditions in the inoculum stage are discussed in detail below. Kinetics and algal physiology were further optimized on a larger scale (production stage) in this multifunctional algology lab, which allows scale-up problems to be solved; which is, in fact, the main goal of any microalgae technology (data not shown).

The results for carotenoids synthesis shown in Table 2 are very promising and are in accordance with those published about autotrophic growth [15]. By applying the hybrid PBR scheme, we were able to achieve a lipid content of about 22% in the production stage (data not shown) compared with about 8–9% achieved in the inoculum stage. In this study, a common approach for the mechanical cell wall disruption and lipid extraction by ultasonification was used. Taking into account that easily degradable carotenoids and proteins could be affected during the extraction procedure, future research should be designed for alternative methods of microalgal cell disruption. In this regard, an innovative approach for enhanced lipid extraction by wall-degrading enzymes is worth investigating [87,88]. Additionally, the recovery of the lipids by the aforementioned method could be enhanced, as was seen previously in the microalgae *Chlorella sorokiniana* and *Nannochloropsis* sp. The quantity of other compounds identified in *S. obliquus* biomass can be easily optimized by using system analysis theory when complex state parameters can be changed to direct the metabolism. Hence, research in the incubation stage is mainly focused on detecting whether there are any promising BAC in extracts of biomass from *S. obliquus*. Further, by using complex stress conditions, we were able to discriminate the concurrent hypothesis and to direct our research to fulfil the ideas of the integral biorefinery approach [8,9,89] Therefore, the chosen unexplored *S. obliquus* 8610 strain has a potential to be developed in the production stage as an ingredient of food or nutraceuticals.

Regarding the antibacterial activity of the extracts *E*1 and *E*2 it could be concluded that single application leads to a strong inhibition of the bacterial growth only in concentrations of 12.5 mg/mL or higher (Table 4). The metabolic activity of the bacteria after treatment with the MIC concentrations was between 1 and 16%, which points to an bacteriostatic effect. Our data are in accordance with the results published by Schuelter et al. [7]. Surprisingly, the addition of low concentrations of the extracts (0.005–0.025 mg/mL) strongly potentiated the antibacterial activity of three clinically used antibacterial drugs—PEN, CIP and ENR—by lowering their effective concentrations two-fold (additive effect) or four-fold (synergism). Both extracts showed similar activity in the combinations (Table 6). Synergism was achieved by the combinations with CIP on all three tested food-borne pathogens, whereas those with PEN on *S. aureus* or ENR on *E. coli* or *S. typhimurium* led to an additive effect. Only the combinations between *E*1 or *E*2 and ENR on *S. aureus* did not potentiate the activity of the chemotherapeutic and the determined effect was “indifference”. These results suggest that microalgae extracts could still be applied as food additives or neutracuticals with health-promoting benefits together with this chemotherapeutic without affecting its antibacterial activity. The combination with OrO is also very promising. Adding 0.01–0.025 mg/mL of the extracts to the oil diminished (from 0.05 to 0.0125% *v*/*v*) the effective concentrations of the latter on all three tested bacterial pathogens four-fold, which is a sign of a synergistic effect. Such combinations have the potential to be used in veterinary practice given the widespread use of oregano oil as an additive in the poultry industry, due to its beneficial effect on body weight and the gut microbiota of the animals [70,73].

It is known that the type of solvent strongly influences the bioactivities of the extracts obtained. The highly non-polar dichloromethane selectively extracts non-polar compounds such as waxes, oils, sterols, chlorophyll and lipids, including fatty acids, etc. [90,91,92,93]. According to published data, the antimicrobial effect of *Scenedesmus* spp. may be due to long-chain free fatty acids since they are one of the most potent antibacterial compounds from microalgae, especially eicosapentaenoic acid [31,52,53,54,94,95,96,97,98], which is abundant in *S. obliquus* [36]. Similarly to Alsenani et al. [99], we did not identify eicosapentaenoic acid in our *S. obliquus* extracts, but there are a number of other long-chain fatty acids with proven antibacterial activity. It is well known from the scientific literature that fatty acids with more than 10 carbon atoms induce lysis of bacteria [52]. The analysis of the chemical composition of our extracts (Table 3) shows the presence of a number of fatty acids with more than 10 carbon atoms. Oleic and linoleic acids, present in microalgal species, have been proved to be active against several human pathogenic bacteria [52,100]. The hydroxylinolenic and trihydroxyoctadecenoic acids detected in our extracts most probably contribute to the antibacterial properties, and hydroxylated fatty acids are also well known for their antimicrobial potential [101]. Other main compounds responsible for the bioactivity of the microalgal species are nonadecane, 7,3′,4′-trimethoxyquercetin and the substances already reported to have antimicrobial activity: 9-octadecadienoic acid (Z), a fatty acid, and butylated hydroxytoluene, which is a derivative of phenol. Other compounds isolated from *S. obliquus* have also been reported to have antimicrobial activity when isolated from other plants: heptadecane, hexadecane, 3-hexadecyloxycarbonyl-5-(2-hydroxyl)-4-methylimidazolium, 2-hexadecenal octasiloxane and the fatty acids 3-hydroxydodecanoic acid and 9,12,15-octadecadienoic acid [30]. Hydroxylated octadecadienoic acid was tentatively identified in the extracts investigated in this study (Table 3). Cepas et al. [102] found that fractions containing among other compounds, such as hexadecatetraenoic acid, (identified in our extracts in a hydroxylated form) exhibit antibacterial and antibiofilm activity. All these published data can partially elucidate the antibacterial activity of our extracts obtained by using dichloromethane as a solvent. Still, there is yet to be a conclusive identification and characterization of the specific metabolites responsible for the antibacterial properties of the extracts *E*1 and *E*2 obtained from biomass of *S. obliquus* strain 8610, which could be an object of future investigations on their antibacterial potential and mechanism of action as individual compounds. The specific mechanisms of the synergistic effect with antibiotics and OrO should also be studied in detail in the future. Regarding OrO, various mechanisms of antibacterial activity of essential oils have been proposed. As a rule, essential oils primarily destabilize the cellular architecture, leading to (i) the breakdown of membrane integrity and increased permeability, which (ii) disrupts many cellular activities; (iii) changes in energy production (membrane-coupled); and (iiii) difficulty in the membrane transport and other metabolic regulatory functions [103]. Due to its lipophilic nature, oregano essential oil easily penetrates through bacterial cell membranes, causing increased membrane permeability leading to the leakage of cellular components and loss of ions [104,105]. The antibacterial effect of essential oils is also linked to reduced membrane potential, disruption of proton pumps and depletion of ATP. These properties of the oregano essential oil most likely contribute to the synergistic effect of the microalgal extracts.

Phenolic compounds from microalgae are both antibacterial and antioxidant. The antioxidant capacity of the extracts can be mainly attributed to the presence of polyphenol structures [91,92]. At high concentrations they can denature cell wall and cell membrane proteins because of the hydrogen bond formed between phenol and protein, causing cell leakage and lysis [31,52,98]. At low concentrations, phenols are reported to affect intracellular enzyme activity, especially of those enzymes associated with energy production [106]. One of the ways in which polyphenols exhibit their antioxidant activity is by chelating metal ions, such as copper ions. The CUPRAC (cupric ion-reducing antioxidant capacity) method has distinct advantages over other electron transfer-based assays because it works at physiological pH [84]. The CUPRAC method is based on the reduction of Cu(II) to Cu(I) [107]. The reduction of copper, as well as the formation of its complexes, is of critical importance since when Cu(II) is in a “free” form it can catalyze the production of free radicals and the development of oxidative processes. The two tested extracts showed different activity with respect to the chelation of copper ions (Figure 2). As Table 3 shows, lutein and zeaxanthin were detected in our extracts, and they have the ability to chelate metal ions [93]. The better ability of *S. obliquus*, cultured under red light, to chelate cupric ions could probably be due to quantitative differences between these compounds, which should be an object of future investigations, and the higher polyphenol content in the biomass collected from SC-PBR2 (Table 2) [84,90,107]. The presence of carotenoids in our extracts suggests that they responsible for the antioxidant capacity, as there is plenty of research in the scientific literature about the antioxidant properties of carotenoids [108,109]. In addition, there have been recent reports about the simultaneous antioxidant and antibacterial potential of fatty acids in plant extracts, and our results are in line with the published data [110,111].

Having in mind the phytochemical analysis of the biomass used for the extraction and of the extracts thereof, we can suppose that the antibacterial potential of our extracts in the tested combinations and the estimated cupric ion-reducing antioxidant capacity are due to the non-polar compounds identified, many of which possess proven antimicrobial (long-chain fatty acids) and/or antioxidant (carotenoids) potential.

## 4. Materials and Methods

### 4.1. Algae Strain, Medium and Cultural Conditions

The algae strain *Scenedesmus obliquus* 8610 was kindly provided by the National Algae Culture Collection—Plovdiv (PACC, Plovdiv, Bulgaria) for the needs of the project KП-06-H37/12 and stored on BBM agar medium in a luminostat chamber at room temperature under low illumination depending on growth and natural light.

The nutrient medium for culturing of *S. obliquus* was modified by using M-8 medium, originally proposed by the authors of [112] in order to reach high-density culture [76]. Kroumov’s medium composition [76] of the modified M-8 was slightly changed and was as follows: (g/L): 3.0 KNO_3_; KH_2_PO_4_ 0.74; CaCl_2_·2H_2_O 0.013; FeSO_4_·7H_2_O 0.13; MgSO_4_·7H_2_O 0.4; NaEDTA·Fe 0.04; and NaHPO_4_ 0.26. Sodium bicarbonate was added as a buffer. This is a three-times concentrated medium allowing fast growth after transferring the inoculum to bigger PBRs in production stage.

The temperature during the cultivation process was 24–28 °C. The light intensity photon flux was achieved by using three 1200 mm LED lamps: T8 SMD, 18 W 1800 lm 6400 K and AC 230 V 50 Hz, behind the small PBRs during inoculum stage. In front of the PBRs a strong-light halogen lamp (approximately 500 W) was used in order to achieve up to the 500 μmol/m^2^/s necessary to stress the cells to synthesize BAC. The halogen lamp stand was constructed to be flexible in order to adjust to the PBRs and to achieve desired illumination. The photoautotrophic growth was under the light–dark period 24 h:0 h. During inoculum stage, *S. obliquus* cells were cultivated in bubble columns with a 8.5 cm inner diameter and 19 cm height. A flow rate between 0.1–0.3/L/L/min with 2–10% CO_2_ content was supplied to the small PBRs. The pH changed because of the growth and CO_2_ supply in the interval between 7.5–9.0. During cultivation, the liquid removed because of sampling and water evaporation was replaced with equivalent amounts of water and medium.

### 4.2. Dry Weight Estimation, Calculation of Specific Growth Rate, Lyophilization of Microalgal Biomass and Preparation of Dichloromethane Extracts

Estimation of dry weight (dw) and specific growth rate was as in standard protocols in biotechnology [11]. Samples of 50 mL withdrawn from PBRs were centrifuged for 10 min at 4000× *g*, harvested and dried at 105 °C until a constant weight was reached. The harvested biomass was freeze-dried (lyophilized) in a vertical freeze dryer (BIOBASE Group, BK-FD18P, Jinan, Shandong, China) according to the protocol published before [7] and the manufacturer instructions for the freeze dryer usage. Briefly, the microalgal biomass was first frozen at −80 °C within the lyophilizer, and then the samples were placed on shelves in a standard chamber under a deep vacuum and dried for 48 h.

The specific growth rate (*μ*) was calculated for the exponential growth phase of the biomass profile (growth curve, see Table 1), which is equal to the balance of the PBR in batch mode (where μ is assumed to be constant):

(2) $$\frac{dX}{dt}=\mu .X$$

(3) $$\int_{X1}^{X2}\frac{dX}{X}=\int_{t1}^{t2}\mu dt$$

Solution of the differential equation after transformation gives the mathematical form of specific growth rate:

(4) $$\mu =\frac{\left(\ln\left(X(2\right)\right)-\ln\left(X\left(1\right)\right))}{t\left(2\right)-t\left(1\right)},$$

where *X*(1) and *X*(2) stand for the biomass concentrations in exponential phase of the growth curve at the time of cultivation *t*(1) and *t*(2), respectively, for the selected time intervals. Time can be chosen from one point of the exponential phase to the other before the culture enters the stationary phase of cell growth or divided into any smaller intervals in the logarithmic phase of growth curve in order to find out the maximum SGR for the given cultivation conditions.

Once again, it must be noted that SGR (*µ*) in the above Equation (4) is a solution of the differential Equation (2) where the key assumption is that *µ* = const in this part of the exponential growth phase for the given period of time and operational conditions. This assumption means that state parameters such as T, pH, light irradiation and macro- and micronutrients of the medium do not limit the growth [8]. Hence, under the above assumption Equation (2) can be solved, and a mathematical form of Equation (4) can be obtained.

Dichloromethane extracts were prepared from the lyophilized microalgal biomass. Briefly, two biomass samples cultured under green light (2.2157 g) and red light (2.0004 g) were extracted with 100 mL of dichloromethane (*x*3) for 15 min each time with ultrasound-assisted extraction to yield 0.1052 g (4.75%) and 0.0858 g (4.29%) of extract, respectively. The extracts were dried and thereafter, the working solutions were prepared in ethanol (96%, #603-002-00-5, Honeywell Specialty Chemicals, Seelze, Germany) in a concentration of 200 mg/mL and dissolved by ultrasonification (ultra-sound bath BIOBASE Group UC-20C, Jinan, China) before use.

### 4.3. Quantitative Determination of Polyphenols in S. obliquus Biomass

The polyphenols were quantitatively determined according the Eur. Ph.8.0. with a slight modification. A total of 0.0200 g of lyophilized microalgae culture was placed in a 50 mL round-bottomed flask and 10 mL of distilled water was added. Then, the sample was heated in an ultrasonic water bath (220 V, 50 Hz, UC-20S, Biobase, Shandong, China) for 30 min. The mixture was filtered through a filter paper. A total of 2.0 mL of the filtrate was diluted to 10.0 mL with water. Then, 2.0 mL of this solution was mixed with 1.0 mL of Folin–Ciocalteu’s phenol reagent (Sigma^®^ Life Science, Steinheim, Germany) and 10.0 mL of water and diluted to 25.0 mL with a 290 g/L solution of sodium carbonate (Honeywell Fluka™, Charlotte, North Carolina, USA). After 30 min, the absorbance at 760 nm was measured using water as the compensation liquid on UV-VIS spectrophotometer (UV-1203, Shimadzu, Japan). The percentage content of total polyphenols was expressed as pyrogallol equivalent [113].

### 4.4. Quantitative Determination of Flavonoids in S. obliquus Biomass

An amount of 0.02 g of the powdered microalgae culture was placed in a 50 mL flask and 10 mL of methanol (Honeywell Speciality Chemicals, Riedel-de Haën, Germany) was added. Then, the sample was heated in an ultrasonic water bath for 30 min; subsequently, it was filtrated in a volumetric flask and diluted to 10 mL with MeOH. Test solution was prepared as follows: 500 µL of solution A was placed in a volumetric flask and diluted to 2 mL with a solution of 20 g/L aluminium chloride (Sigma-Aldrich, Switzerland) in methanol. Compensation solution consisted of 500 µL of solution A placed in a volumetric flask and diluted to 20.0 mL with MeOH. After exactly 15 min, the absorbance of the test solution was measured at 420 nm by comparison with the compensation solution. The total flavonoid content was expressed as hyperoside [114].

### 4.5. Quantitative Determination of Chlorophylls and Carotenoids in S. obliquus Biomass

The method of Gonçalves et al. [13] was applied for quantitative determination of chlorophylls and carotenoids. A total of 0.01 g of lyophilized microalgae culture (LMC) was placed in an Erlenmeyer flask, and 5 mL of acetone (Honeywell Speciality Chemicals, Riedel-de Haën, Germany) solution (80%) was added, maintaining the sample in ultrasonic water bath for 20 min with subsequent centrifugation (3000× *g*, 8 min). The supernatant absorbance was measured at wavelengths of 470, 646.8 and 663.2 nm against a blank consisting of acetone (80%). The concentration of pigments (mg g LMC^−1^) was calculated using Equations (5)–(7) [115]:

Chl*a* = 12.25 × *A*_663.2_ − 2.79 × *A*_646.8_ (5)

Chl*b* = 21.50 × *A*_646.8_ − 5.10 × *A*_663.2_ (6)

TC = (1000 × *A*470 − 1.82 × Chl*a* − 85.02 × Chl*b*)/198 (7)

where Chl*a*: chlorophyll *a*; Chl*b*: chlorophyll *b*; TC: total carotenoids; *A*_646.8_: absorbance in 646.8 nm; *A*_663.2_: absorbance in 663.2 nm; and A_470_: absorbance in 470 nm.

### 4.6. Quantitative Determination of Lipids in S. obliquus Biomass

The total lipids were quantified according the method applied by Carpio et al. [116], and chloroform–methanol mixture (2:1, *v*/*v*) was used in three (3) steps [116]. At each step, 0.01 g of algae powder sample was soaked in 5 mL of solvent for 8 h. Then, the mixture was centrifuged at 4000× *g* for 5 min. The solvent–lipid phase was carefully transferred to a pre-weighted aluminium dish and the solvent was evaporated to dryness under vacuum. The total lipids were determined gravimetrically; the percent of total lipids was calculated as the percent ratio of dry weight of extracted lipids to dry algae biomass used. Reported values were averages of three measurements.

### 4.7. UHPLC–HRMS

To assess the composition of the tested dichloromethane extracts, nontargeted metabolic profiling was performed by UHPLC-HRMS in Full scan-ddMS2/Top 5 scan mode. Mass analyses were carried out on a Q Exactive Plus mass spectrometer (Thermo Fisher Scientific, Inc., Waltham, MA, USA) equipped with a heated electrospray ionization (HESI-II) probe (Thermo Scientific, Inc., Waltham, MA, USA). Briefly, taking into consideration the mass range for survey full scan 100−1000 Da, stepped collision energy (NCE) was set at 20, 40, 70 for data-dependant (dd) MS^2^ scans [117].

UHPLC separation was carried out on a reversed phase column Kromasil EternityXT C18 (1.8 µm, 2.1 × 100 mm) column maintained at 40 °C. The binary mobile phase consisted of A: 0.1% formic acid in water and B: 0.1% formic acid in acetonitrile. The run time was 33 min. The following gradient was used: the mobile phase was held at 5% B for 1 min, gradually turned to 30% B over 19 min, increased gradually to 50% B over 5 min, increased gradually to 70% B over 5 min and finally increased gradually to 95% over 3 min. The system was then turned to the initial condition of 5% B and equilibrated over 4 min. Mass spectrometry analyses were carried out on a Q Exactive Plus mass spectrometer (ThermoFisher Scientific, Inc., Waltham, MA, USA) equipped with a heated electrospray ionization (HESI-II) probe (ThermoScientific, Inc., Waltham, MA, USA). The tune parameters were as follows: spray voltage 3.5 kV (+) and 2.5 kV (−); sheath gas flow rate 38; auxiliary gas flow rate 12; spare gas flow rate 0; capillary temperature 320 °C; probe heater temperature 320 °C; and S-lens RF level 50. Acquisition was acquired at full-scan MS (FS-MS) and data-dependent-MS2 (DD-MS2) modes. FS-MS spectra over the *m*/*z* range from 100 to 1000 were acquired in negative and positive ionization modes at a resolution of 70,000. Other instrument parameters for FS-MS mode were set as follows: automatic gain control (AGC) target 1e6, maximum ion time (IT) 50 ms, number of scan ranges 1. For DD-MS2, instrument parameters were as follows: microscans 1, resolution 17,500, AGC target 1e5, maximum IT 50 ms, MSX count 1, Top5, isolation window 2.0 *m/z*, stepped normalized collision energy (NCE) 10, 30, 60. Data acquisition and processing were carried out with Xcalibur 4.2 software (ThermoScientific, Inc., Waltham, MA, USA) [117].

### 4.8. Distillation of Oregano Oil

The *Origanum vulgare* plant originates from the area of Panagyurishte, Sredna Gora Mountains, Bulgaria. The essential oil was obtained by distillation as described before [64] using a Clevenger apparatus and was stored in the fridge (5–7 °C). The chemical composition of the extract used in the experiments in this study was published before [64].

### 4.9. Bacterial Strains and Culture Conditions

The following three bacterial strains were selected for determination of antimicrobial susceptibility testing in our study: *Staphylococcus aureus* (ATCC^®^ 29213^TM^, American Type Cell Culture Collection, Manassas, VA, USA), *Escherichia coli (*ATCC^®^ 35218^TM^, Manassas, Virginia, USA) and *Salmonella typhimurium* (Strain 123, Collection of the Stephan Angeloff Institute of Microbiology, Bulgarian Academy of Sciences). The strains were maintained in Trypticase Soy Agar/Broth (TSA/TSB, Himedia, India) at 37 °C under aerobic conditions. All experiments for determination of MIC were performed in Mueller Hinton broth (MHB, #M0405B, Thermo Scientific-Oxoid, Hampshire, UK).

### 4.10. Determination of Minimal Inhibitory Concentrations

The minimal inhibitory concentrations (MIC) of both microalgal extracts were determined with the broth microdilution method (BMD) according to ISO 20776/1-2006 [118]. The working solutions of the extracts were prepared in ethanol in concentration of 200 mg/mL and dissolved by ultrasonification as described in point 4.1. Briefly, two-fold serial dilutions of the extracts ranging from 0.06 to 12.5 mg/mL were prepared in 96-well plates in a volume of 50 µL. Each concentration was repeated three-fold. MHB was used as diluent and served as negative control. An overnight liquid bacterial culture was diluted in MHB to a bacterial suspension with optical density 10^8^ CFU/mL (OD_600_) and brought thereafter to a final density of 5 × 10^5^ CFU/mL. An equivalent volume (50 µL) of the second bacterial suspension (final bacterial density: 5x10^4^ CFU/mL) was added to each well of the plates except the negative control. The plates were incubated 24 h at 37 °C. The lowest drug concentration that prevented visible bacterial growth was determined as MIC. Benzathine benzylpenicillin (PEN, #B0500000, Merck KGaA, Darmstadt, Germany), ciprofloxacin (CIP, Ciproflav: 10 mg/mL, Polfa S.A.Warsaw Pharmaceutical Works, Starogard Gdański, Poland) and enrofloxacin (ENR, Interflox oral: 100 mg/mL, Interchemie Werken “De Adelaar” B.V., Venray, The Netherlands) were used as referent antibiotics (positive controls). They were applied in the following concentration ranges: 0.004–4 mg/L (PEN), 0.002–2 mg/L (CIP) and 0.003–0.4 mg/L (ENR). The requirements of EUCAST (European Committee on Antimicrobial Susceptibility Testing) for their MICs were followed for discussing the results [119]. PBS served as a negative control.

### 4.11. Checkerboard Assay

The checkerboard BMD test was used for the in vitro evaluation of combinations between the microalgal extracts, on one hand, and clinically approved chemotherapeutics/antibiotics or oregano essential oil on the other. Each extract was mixed in a 96-well plate with CIP, ENR or OrO for all strains or with PEN G for *S. aureus*. The serial dilutions were prepared in a two-dimensional fashion to include all combinations (42/plate in our case) within a specified clinically relevant range for the respective chemotherapeutic or antibiotic. The BMD test was performed as described above. MICs were determined after 24 h of incubation.

The scheme for the combination of the microalgal extracts and PEN, CIP, ENR or OrO followed the recommendations of the checkerboard assay [82]. For the combinations, each extract was applied at concentrations ranging between 0.005 and 0.39 mg/mL. The concentration range of PEN and the fluoroquinolones were as follows: (1) PEN on *S. aureus*—from 0.0156 up to 1 mg/L; (2) CIP on *S. aureus*—from 0.0156 up to 2 mg/L; CIP on *E. coli* and *S. typhimurium*—from 0.00156 up to 0.2 mg/L and 3) ENR on all three strains—from 0.00625 up to 0.4 mg/L. OrO was applied in concentrations between 0.00156 and 0.2%. All tested drugs and extracts were applied in two-fold serial dilutions.

The FIC (fractional inhibitory concentration) methodology was applied for evaluation of the combination effects. The FICs were calculated by comparing the MIC of each drug/extract alone to the MIC of that drug/extract in the combination (MIC_C_). The FICs were calculated and interpreted as follows [120]:

- Step 1

(8) $$FIC\;\left(A\right)=\frac{MIC_{C}\;\left(A\right)}{MIC\;\left(A\right)}$$

(9) $$FIC\;\left(B\right)=\frac{MIC_{C}\;\left(B\right)}{MIC\;\left(B\right)},$$

where *FIC* means fractional inhibitory concentration, *A* stands for drug A (microalgal extracts), and *B* is drug B (PEN, CIP, ENR or OrO).

- Step 2

(10) $$\sum FIC=FIC\;\left(A\right)+FIC\left(B\right)$$

Synergy was defined as *ƩFIC* ≤ 0.5, indifference—as 0.5 < Ʃ*FIC* ≤ 4 and antagonism—as Ʃ*FIC* > 4. Some investigators [120] consider compounds additive when 0.5 < Ʃ*FIC* ≤ 1, which was adopted for this study.

### 4.12. Metabolic (Cell Redox, Respiratory and Dehydrogenase) Activity Assay

The cell redox (dehydrogenase) activity of treated bacteria was measured for both the MBD test and the checkerboard assay following the protocol of Wang et al. [121]. Briefly, at the end of the incubation period MTT dye (5 mg/mL in PBS) was added to each sample to obtain final concentration of 0.25 mg/mL and mixed thoroughly. Plates were incubated for 120 min at 37 °C. During that incubation period the MTT is reduced by the membrane-located bacterial enzyme NADH–ubiquinone reductase (H^+^-translocation) to non-soluble violet crystals of formazan. The latter were dissolved with an equivalent volume of organic solvent (5% formic acid in 2-propanol). The absorbance was measured at *λ* = 550/690_ref_ nm (Absorbance Microplate Reader L*x*800, BioTek Instruments Inc., Santa Clara, CA, USA) against a blank solution containing the respective volumes of MHB, MTT and solvent. The inhibition in the metabolic activity of the treated bacteria was calculated as percent of the untreated control.

### 4.13. Cell Viability Assay

The cell viability of normal mice fibroblasts (CCL-1^TM^, NCTC clone 929, American Type Culture Collection—ATCC, Manassas, VA, USA) was evaluated according to ISO 10993-5, Annex C [86]. The cells were cultured at 37 °C under sterile conditions and humidified atmosphere in a CO_2_ incubator (Panasonic MCO-18AC, Kadoma, Osaka, Japan) supplied continuously with 5% CO_2_. Cells were maintained in the culture medium MEM (#MEM-A, Capricorn^®^, Munich, Germany) with addition of 10% heat inactivated horse serum (#HOS-1A, Capricorn^®^, Munich, Germany) and 2 mM of L-glutamine (#G7513, Sigma-Aldrich, Steinheim, Germany). Cells were seeded in 96-well plates in concentration 1 × 10^4^/100 µL medium/well as recommended in ISO 10993-5, Annex C [86]. The tested concentrations of the extracts varied from 0.02 up to 1 mg/mL in two-fold serial dilutions. The incubation period was 72 h. The cell viability was measured on a microplate reader ELx800 (BioTek Instruments, Inc., Winooski, VT, USA) at *λ* = 540 nm/_ref_690 nm.

### 4.14. Cupric Ion-Reducing Antioxidant Capacity (CUPRAC) Assay

The assay was performed according to the method of Apak et al. [122]. The CUPRAC reaction of Cu(II)–neocuproine complex with antioxidants results in a change from blue to yellow/orange due to Cu(I)–neocuproine chelate (λ_max_ = 450 nm). The following solutions were as follows:

(1) 10 mM of CuCl_2_ in distilled H_2_O;
(2) 1.0 M of ammonium acetate buffer; pH7;
(3) 7.5 mM of neocuproine (NC) in 96% ethanol.

The common reaction mixture was prepared in the following arrangement: solution 1 part (1): 1 part (2): 1 part (3).

Stock solutions of the *S. obliquus* extracts (50 mg/mL) were diluted in PBS to prepare samples with different concentrations ranging from 0.15 up to 2.5 mg/mL. The standard curve was prepared based on the absorbance of Trolox in concentrations varying from 0.05 mM up to 1 mM. Results were expressed as mM of Trolox equivalent per g extract. In addition, the antioxidant capacity of both extracts to reduce Cu (II) ions was expressed as a ratio between the EC_50_ values of the Trolox and the microalgal extract as follows: TEAC = EC_50_ of Trolox [mg/mL]/EC_50_ of sample [mg/mL] [85]. The higher TEAC value means higher antioxidant capacity for reducing Cu (II) ions.

### 4.15. Mathematical Modelling of Redox-Modulating Capacities

The median effective concentrations EC_50_ of the microalgae extracts were calculated by adapting an algorithm published elsewhere [123,124]. In this work we used the developed program for non-linear identification procedure in the MAPLE^®^ software. Details can be found in [123,124]. The same median dose model was applied as presented in Equation (11):

(11) FaFu=DoseDmm

where *F*_a_ stands for affected fraction; *F*_u_ stands for unaffected fraction (1 − *F*_a_) = *F*_u_; Dose is the applied concentration of the microalgae extract; *D_m_* represents the median-effect dose (in our case *D*_m_ = EC_50_), and *m* is the slope of the median-effect plot.

### 4.16. Statistics

All experiments were carried out in triplicate. Each concentration was repeated two-fold in the MBD and bacterial metabolic activity tests, and four-fold in the cell viability assay. The statistical evaluation of the data was performed with the two independent sample Student’s t-test for data from the phytochemical analysis of microalgal biomass or one-way ANOVA (GraphPad Prism software, version 6.0.0 for Windows, San Diego, CA USA) for the in vitro cytotoxicity data. A *p* value < 0.05 was considered as statistically significant.

## 5. Conclusions

The present work directed us to witness how complex efforts in a multifunctional lab can be accomplished in the aspect of the integral biorefinery concept. The inoculum and production stages served for checking the hypotheses of achieving maximum CO_2_ utilization, which is useful for the efforts to minimize CO_2_ emissions from waste industrial gases and as a result to obtain high biomass concentration. The higher value of the latter was achieved in SC-PBR1 (6.95 g/L). The internal and external colour LED combination is very promising when applied in the production stage in terms of the synthesis of secondary metabolites. Concerning the production of biopigments, the application of internal green or red light resulted in total carotenoids between 5 and 5.5 mg/g dw in SC-PBRs 1 and 2, which is a promising result in the state of the art for *Scenedesmus* spp. The application of engineering solutions to create innovative PBRs in the inoculum stage resulted in quality biomass of the unexplored *Scenedesmus obliquus* strain, representing a source for extracts and compounds with significant antibacterial potential in combination with clinically applied antibiotics and oregano oil, as well as promising antioxidant properties. The investigated extracts can serve as basis for development of health-promoting nutraceuticals for use as additives to a classical diet or antibacterial drugs in the poultry industry.

## Figures and Tables

**Table 1:** Growth of the microalgal biomass in SC-PBR1 and SC-PBR2 under given conditions in the inoculum stage.

Cultivation Time [Days]	Biomass Concentration—Dry Weight [g/L]	
	SC-PBR1 (Green Light)	SC-PBR2 (Red Light)
0	0.32	0.41
6	2.85	2.18
14	3.36	2.61
21	4.32	4.78
29	6.95	5.87
36	5.72	5.55

**Table 2:** Phytochemical analysis of lyophilized microalgal biomass obtained from *S. obliquus* cultured under different light conditions.

Microalgal Strain	Flavonoids % (g/100 g dw)	Polyphenols % (g/100 g dw)	Chlorophyll a mg/g dw	Chlorophyll b mg/g dw	Total Carotenoids mg/g dw	Lipids % (g/100 g dw)
*S. obliquus*, green light, SC-PBR1	0.85 ± 0.11	1.49 ± 0.15	16.38 ± 0.44	6.73 ± 0.21	5.49 ± 0.18	8.88 ± 0.12
*S. obliquus*, red light, SC-PBR2	0.61 ± 0.09	2.15 ± 0.07	16.80 ± 0.28	7.88 ± 0.29	5.14 ± 0.15	8.97 ± 0.13
Statistical analysis (*p*-value) *	0.0353	0.0021	0.2382	0.0053	0.0671	0.4541

Legend: dw–dry weight; * A *p*-value < 0.05 was considered statistically significant.

**Table 3:** UHPLC-HRMS profiling of *S. oblicuus* dichloromethane extracts.

№	Tentatively Annotated Compound	Molecular Formula	Exact Mass [M + H]^+^	Fragmentation Pattern (Relative Abundance)	*t*_R (min)_
**1**	carotene	C_40_H_56_	537.4455	537.4359 (100), 406.3410 (9.3), 322.2479 (3.4), 198.1847 (9.9), 114.0914 (38.6), 95.0858 (1.6), 69.0705 (10.3)	11.26
**2**	canthaxanthin	C_40_H_52_O_2_	565.4040	565.4030	22.60
**3**	hydroxyechineone	C_40_H_54_O_2_	567.4197	567.4184 (100), 549.4068 (11.9), 169.1007 (20.9), 147.1163 (8.8), 145.1008 (45.0), 119.0857 (60.5), 105.0701 (83.24), 93.0704 (47.5), 69.0705 (8.3)	22.90
**4**	lutein/zeaxanthin	C_40_H_56_O_2_	569.4353	569.4304	22.70
**5**	neoxanthin/violaxanthin	C_40_H_56_O_4_	601.4251	601.4150	22.62
	**Tentatively Annotated Compound**	**Molecular Formula**	**Exact Mass** **[M − H]^−^**	**Fragmentation Pattern** **(Relative Abundance)**	***t*_R (min)_**
**6**	hydroxyhexadecatetraenoic acid	C_16_H_24_O_3_	263.1658	263.1656 (81.22), 245.1541 (23.47), 242.9861 (27.09), 219.1750 (13.63), 205.1228 (36.81), 201.1643 (100), 173.1328 (5.00), 161.1325 (67.24), 159.165 (3.61), 147.1169 (25.76), 133.1002 (3.25), 107.0853 (23.33), 97.0643 (4.17), 71.0486 (39.93), 59.0123 (2.58), 57.0329 (12.89)	13.15
**7**	hydroxyhexadecatrienoic acid	C_16_H_26_O_3_	265.1811	265.1811 (100), 247.1703 (70.26), 229.1596 (0.80), 207.1385 (94.60), 181.1222 (0.97), 163.1480 (5.12), 149.1324 (3.25), 83.0485 (1.10), 71.0487 (9.99), 59.0123 (12.30), 57.0330 (1.31)	13.78
**8**	hydroxyhexadecadienoic acid	C_16_H_28_O_3_	267.1968	267.1968 (100), 249.1862 (34.79), 216.9886 (1.37), 205.1955 (1.13), 167.1068 (54.06), 149.0961 (3.35), 59.0123 (7.73)	15.04
**9**	octadecatetraenoic acid (stearidonic acid)	C_18_H_28_O_2_	275.2015	275.2015 (100), 231.2119 (4.30), 177.1634 (2.06), 59.0123 (4.58)	15.94
**10**	dihydroxyhexadecapentaenoic acid	C_16_H_22_O_4_	277.1451	277.1447 (79.51), 259.1348 (5.20), 249.2476 (1.29), 233.1526 (3.73), 221.1182 (41.22), 177.1275 (63.60), 161.0961 (14.63), 149.0961 (16.73), 135.0802 (100), 121.0646 (20.28), 97.0644 (64.25), 95.0487 (13.82), 71.0487 (31.35), 59.0123 (21.09), 57.0330 (9.94)	
**11**	dihydroxyhexadecatetraenoic acid	C_16_H_24_O_4_	279.1609	279.1595 (19.30), 261.1496 (13.77), 207.1021 (85.26), 181.0863 (29.95), 163.1113 (2.64), 157.0860 (65.28), 139.0750 (6.96), 121.0645 (51.56), 97.0644 (100), 95.0488 (10.09), 83.0487 (10.53), 65.0381 (35.25), 59.0123 (7.19)	10.24
**12**	hydroxyoctadecatrienoic acid (hydroxylinolenic acid)	C_18_H_30_O_3_	293.2127	293.2126 (80.24), 275.2017 (100), 231.2111 (5.27), 183.1019 (0.87), 171.1018 (3.33), 121.1008 (1.76), 71.0486 (2.13)	15.92
**13**	hydroxyoctadecatrienoic acid isomer	C_18_H_30_O_3_	293.2125	293.2125 (100), 275.2021 (8.55), 231.2133 (0.48), 223.1336 (14.32), 195.1383 (12.33), 179.1431 (0.79), 111.0799 (0.55), 87.0948 (0.48), 71.0035 (0.60), 59.0121 (0.58)	16.12
**14**	dihydroxyoctadecapentaenoic acid	C_18_H_26_O_4_	305.1767	305.1763 (100), 287.1656 (9.13), 269.1551 (1.59), 233.1180 (1.94), 221.1179 (2.00), 205.1594 (5.28), 185.1177 (2.68), 163.1124 (1.62), 151.1119 (1.92), 135.0803 (98.45), 125.0959 (19.2), 97.0644 (16.61), 79.0538 (11.99), 57.0330 (2.23)	13.25
**15**	dihydroxyoctadecatetraenoic acid	C_18_H_28_O_4_	307.1923	307.1921 (37.36), 289.1814 (23.68), 235.1338 (100), 211.1335 (38.20), 185.1175 (87.44), 167.1442 (0.73), 141.1270 (1.03), 137.0961 (2.41), 125.0959 (33.31), 121.0645 (91.93), 97.0644 (64.24), 83.0488 (0.99), 65.0381 (42.23), 71.0487 (32.56), 57.0329 (2.65)	12.30
**16**	dihydroxyoctadecatrienoic acid	C_18_H_30_O_4_	309.2080	309.2076 (100), 291.1970 (59.85), 273.1873 (8.87), 229.1957 (4.12), 263.2017 (4.89), 251.1652 (56.36), 225.1493 (37.13), 209.1541 (83.09), 197.1175 (41.33), 175.1483 (1.44), 135.1164 (1.17), 11,100,799 (14.94), 97.0641 (5.35), 83.0487 (2.08), 71.0486 (7.65), 57.0331 (0.96)	14.03
**17**	dihydroxyoctadecadienoic acid	C_18_H_32_O_4_	311.2237	311.2232 (100), 293.2125 (15.19),275.2009 (2.93), 249.2224 (0.56), 227.2135 (0.28), 211.1335 (15.79), 197.1177 (8.26), 171.1017 (17.25), 139.1116 (3.32), 129.0907 (7.02), 113.0956 (2.08), 99.0798 (1.62), 83.0488 (0.42), 57.0330 (1.32)	15.11
**18**	trihydroxyoctadecatetraenoic acid	C_18_H_28_O_5_	323.1873	323.1861 (61.84), 305.1762 (68.47), 287.1656 (58.74), 243.1755 (9.49), 237.1495 (89.71), 209.1178 (56.60), 171.1013 (14.89), 151.0754 (10.46), 135.0801 (11.94), 125.0958 (11.83), 113.0594 (100), 95.0487 (57.09), 83.0488 (26.95), 71.0487 (28.29), 57.0332 (12.39)	12.40
**19**	trihydroxyoctadecatrienoic acid	C_18_H_30_O_5_	325.2017	325.2017 (24.32), 307.1913 (19.90), 289.1813 (83.61), 245.1910 (6.28), 237.1495 (100), 211.1335 (1.82), 201.1126 (49.71), 197.1170 (2.71), 171.1021 (2.09), 123.0804 (3.10), 109.0646 (6.46), 83.0486 (3.38), 57.0331 (9.74)	11.29
**20**	trihydroxyoctadecenoic acid	C_18_H_34_O_5_	329.2342	329.2337 (100), 311.2227 (0.90), 293.2119 (0.92), 268.9841 (0.37), 229.1442 (6.79), 211.1337 (10.37), 183.1380 (0.92), 171.1017 (26.15), 157.1231 (1.46), 139.1116 (9.08), 127.1115 (3.29), 99.0801 (4.46), 69.0964 (0.89), 87.0329 (1.24)	10.68

**Table 4:** Antibacterial activity against food-borne pathogens of dichloromethane extracts obtained from lyophilized biomass of *S. obliquus* cultured in SC-PBR1 and SC-PBR2—minimal inhibitory concentrations of the extracts and metabolic activity of the bacteria.

Extracts of Lyophilized Biomass from:	*S. aureus*		*E. coli*		*S. typhimurium*	
	MIC [mg/mL]	DEHA [%]	MIC [mg/mL]	DEHA [%]	MIC [mg/mL]	DEHA [%]
*S. obliquus*, green light (E1)	>12.5	-	12.5	16 ± 0.31	12.5	5.2 ± 0.7
*S. obliquus*, red light (E2)	>12.5	-	12.5	10.4 ± 0.28	12.5	1.09 ± 0.14

Legend: MIC—minimal inhibitory concentration, DEHA—dehydrogenase (metabolic) activity of the treated bacteria.

**Table 5:** Minimal inhibitory concentrations [mg/L] of PEN; CIP, ENR and OrO after single application.

Bacterial Species	Minimal Inhibitory Concentrations			
	[mg/L]			[%]
	PEN *	CIP **	ENR ***	OrO ****
*S. aureus*	0.125	0.25	0.05	0.05 ^§^
*E. coli*	-	0.0125	0.0125	0.05 ^§^
*S. typhimurium*	-	0.05	0.05	0.05

Legend: * Penicillin, ** Ciprofloxacin, *** Enrofloxacin, **** Oregano essential oil, ^§^ Data published in [64] and confirmed here.

**Table 6:** Combination effects between penicillin, fluoroquinolones or essential oregano oil and extracts from lyophilized biomass of *S. obliquus* cultured under combination of white(external):green(internal) *E*1 and white(external):red(internal) *E*2 light conditions.

Strain	AB/CT/OrO	Extract	MIC_C-extract_	MIC_C-AB/CT/OrO_	FIC_extract_	FIC_AB/CT/OrO_	∑FIC	Effect
*S. aureus*	PEN	E1	0.01	0.0625	0.0008	0.5	0.5008	Additive
		E2	0.01	0.0625	0.0008	0.5	0.5008	Additive
	CIP	E1	0.01	0.0625	0.0008	0.25	0.2508	Synergism
		E2	0.01	0.0625	0.0008	0.25	0.2508	Synergism
	ENR	E1	0.01	0.05	0.0008	1	1.0008	Indifference
		E2	0.01	0.05	0.0008	1	1.0008	Indifference
	OrO	E1	0.005	0.025	0.0004	0.5	0.5008	Additive
			0.025	0.0125	0.002	0.25	0.252	Synergism
		E2	0.01	0.025	0.0008	0.5	0.5008	Additive
*E. coli*	CIP	E1	0.01	0.003125	0.0008	0.5	0.5008	Synergism
		E2	0.01	0.003125	0.0008	0.5	0.5008	Synergism
	ENR	E1	0.01	0.00625	0.0008	0.5	0.5008	Additive
		E2	0.01	0.00625	0.0008	0.5	0.5008	Additive
	OrO	E1	0.005	0.025	0.0004	0.5	0.5004	Additive
			0.025	0.0125	0.002	0.25	0.252	Synergism
		E2	0.005	0.025	0.0004	0.5	0.5004	Additive
			0.01	0.0125	0.0008	0.25	0.5008	Synergism
*S. typhimurium*	CIP	E1	0.01	0.0125	0.0008	0.25	0.2508	Synergism
		E2	0.01	0.0125	0.0008	0.25	0.2508	Synergism
	ENR	E1	0.01	0.025	0.0008	0.5	0.5008	Additive
		E2	0.01	0.025	0.0008	0.5	0.5008	Additive
	OrO	E1	0.005	0.025	0.0004	0.5	0.5004	Additive
			0.025	0.0125	0.002	0.25	0.252	Synergism
		E2	0.01	0.0125	0.0008	0.25	0.2508	Synergism

Legend: AB—antibiotic, concentration [mg/L]; CT—chemotherapeutic, concentration [mg/L]; OrO—oregano essential oil, concentration [%]; Extract concentration is presented in [mg/mL]; MIC_C_—minimal inhibitory concentration in the combination; FIC—fractional inhibitory concentration.

## Data Availability

Research data can be obtained from the authors by e-mail.

## Supplementary Materials

The following supporting information can be downloaded online, Figure S1: (+) ESI-MS/MS (up) and (−) ESI-MS/MS (down) spectra of carotene (1)., Figure S2: Monoisotopic profile of hydroxyechineone (3) in Full MS spectrum (up); (+) ESI-MS/MS spectrum (down)., Figure S3: Monoisotopic profile of canthaxanthine (2) in Full MS spectrum, Figure S4: (−) ESI-MS/MS spectrum of octadecatetraenoic acid (10), Figure S5: (−) ESI-MS/MS spectrum of hydroxyoctadecatrienoic (12), Figure S6: (−) ESI-MS/MS spectrum of dihydroxyoctadecapentaenoic acid (14), Figure S7: (−) ESI-MS/MS spectrum of trihydroxyoctadecatetraenoic acid (18), Figure S8: (−) ESI-MS/MS spectrum of hydroxyhexadecatetraenoic acid (6).

## Sample Availability

Samples of the lyophilized microalgal biomass and the extracts are available from the authors.
