# Supplementary material for: Antimicrobial and Antioxidant Potential of Scenedesmus obliquus Microalgae in the Context of Integral Biorefinery Concept
Source: Molecules. 2022 Jan 14;27(2):519. doi: 10.3390/molecules27020519 (PMC8778625; doi:10.3390/molecules27020519)
Supplement: Supplementary file 1 [file molecules-27-00519-s001.zip › molecules-1512292-supplementary.pdf]

Supplemental material

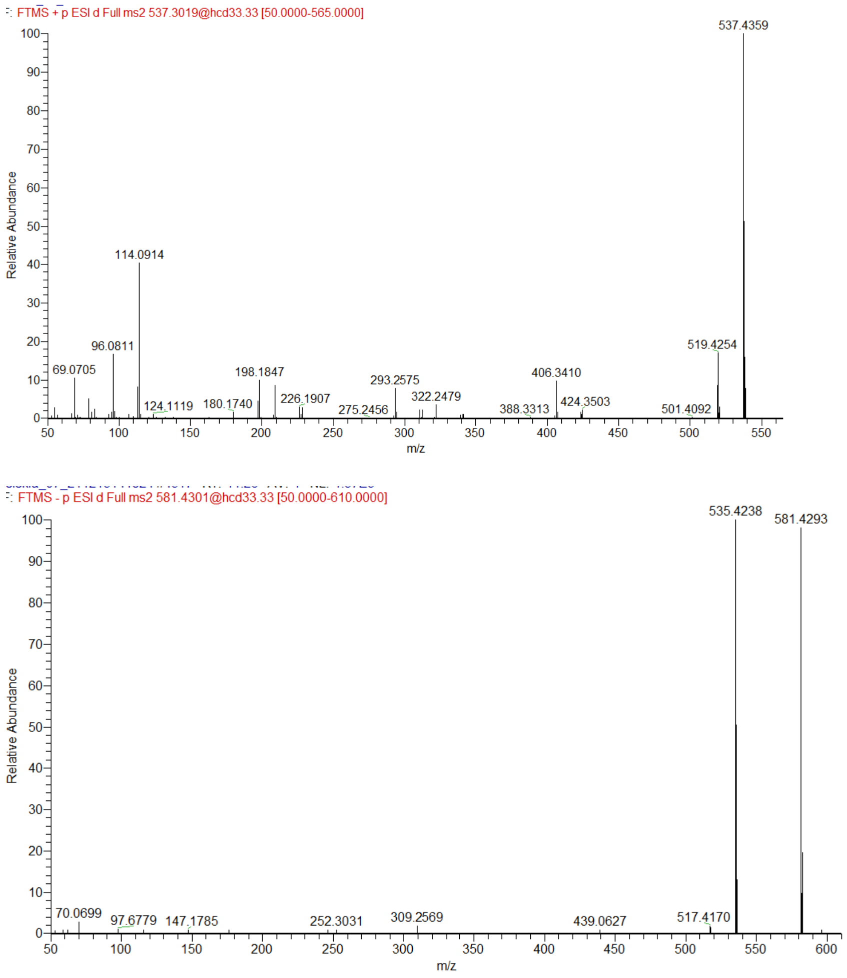

Figure S1. (+) ESI-MS/MS (up) and (-) ESI-MS/MS (down) spectra of carotene (**1**).

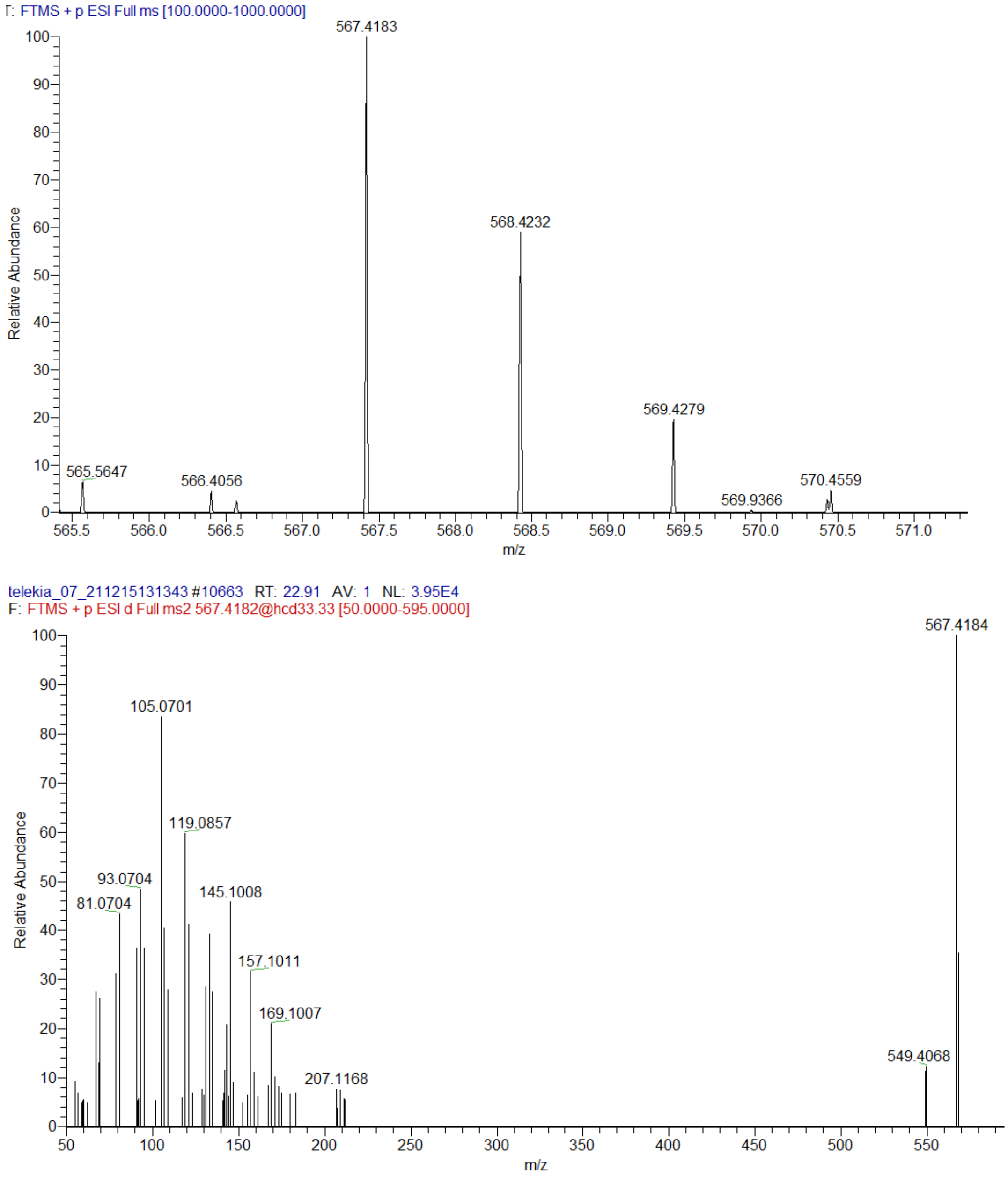

Figure S2. Monoisotopic profile of hydroxyechineone (**3**) in Full MS spectrum (up); (+) ESI-MS/MS spectrum (down).

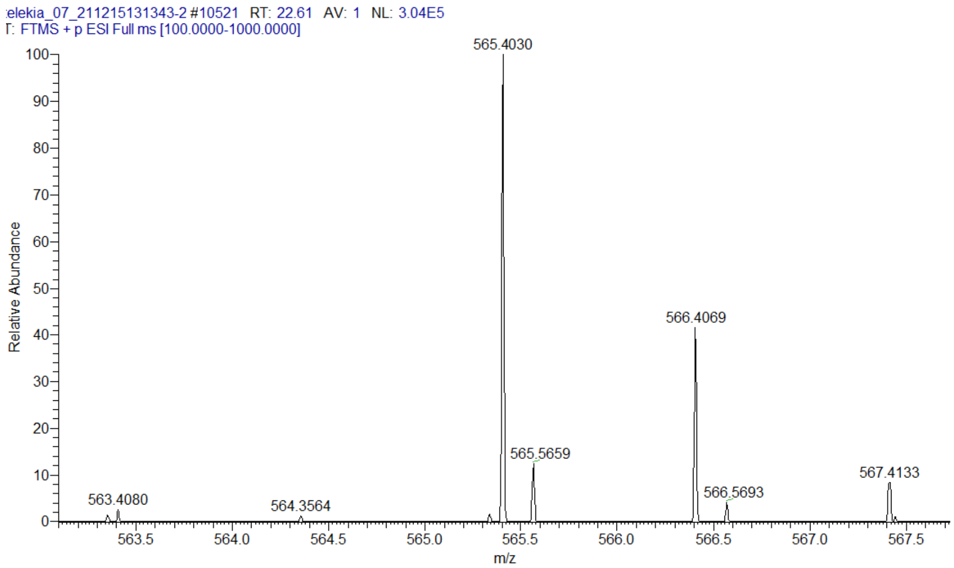

Figure S3. Monoisotopic profile of canthaxanthine (**2**) in Full MS spectrum.

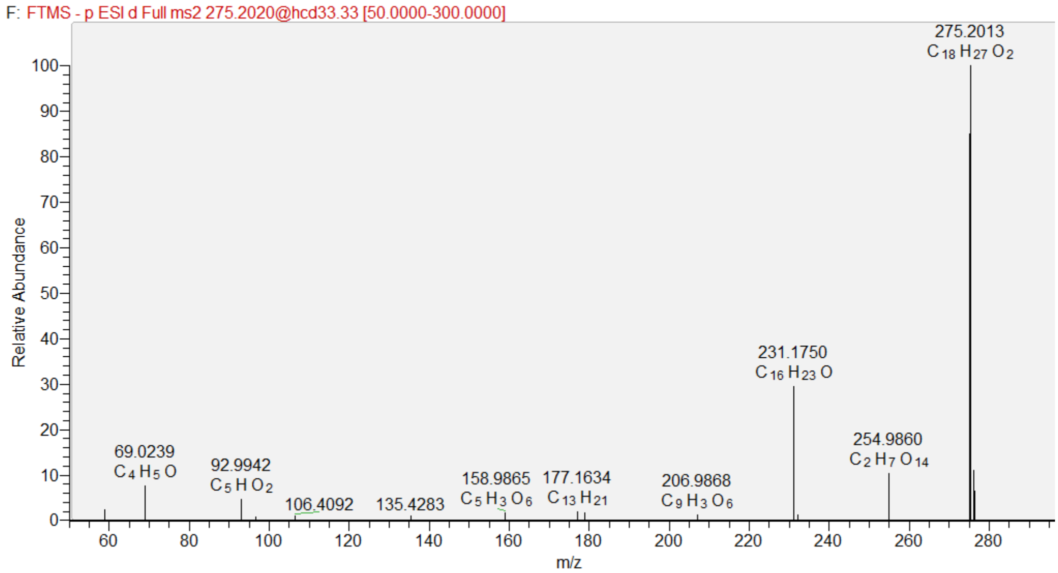

Figure S4. (-) ESI-MS/MS spectrum of octadecatetraenoic acid (**10**).

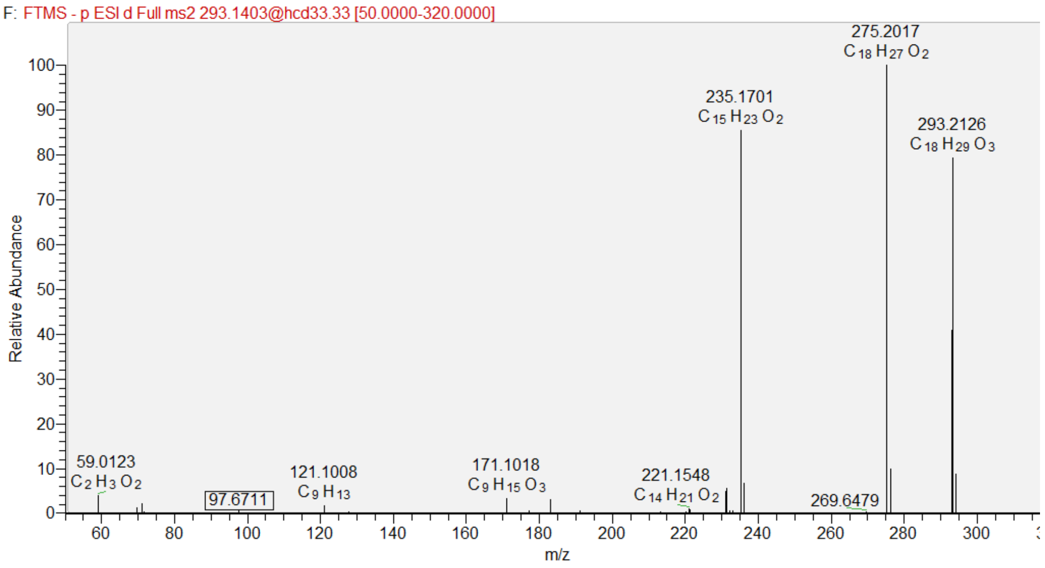

Figure S5. (-) ESI-MS/MS spectrum of hydroxyoctadecatrienoic (**12**).

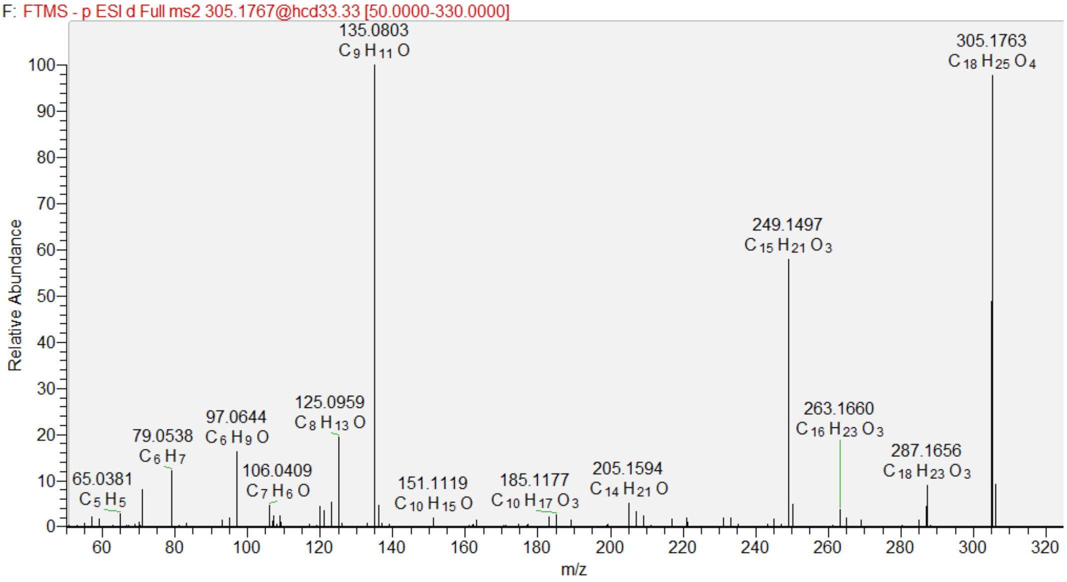

Figure S6. (-) ESI-MS/MS spectrum of dihydroxyoctadecapentaenoic acid (**14**).

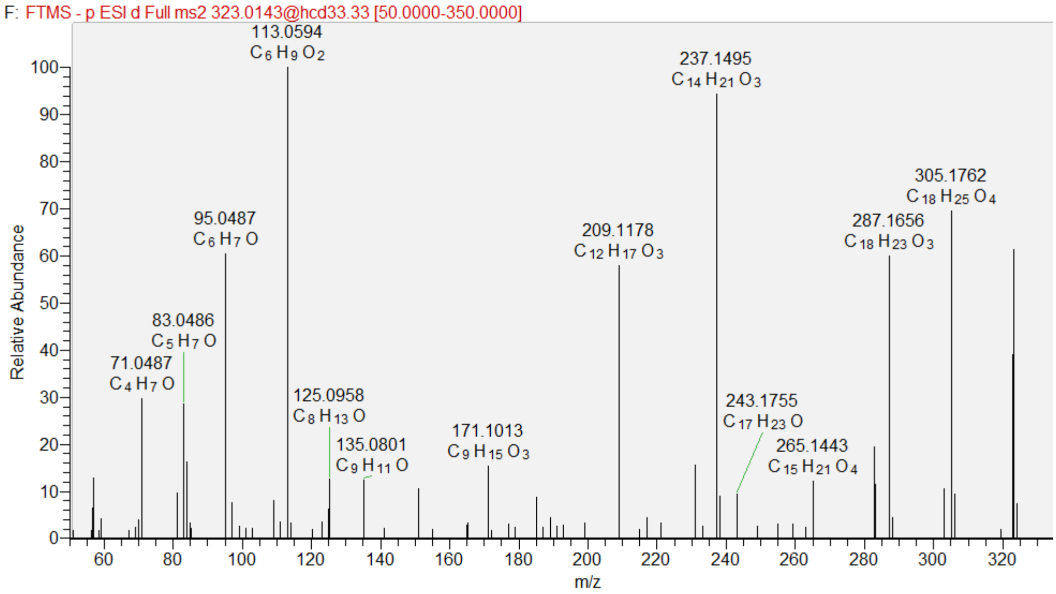

Figure S7. (-) ESI-MS/MS spectrum of trihydroxyoctadecatetraenoic acid (**18**).

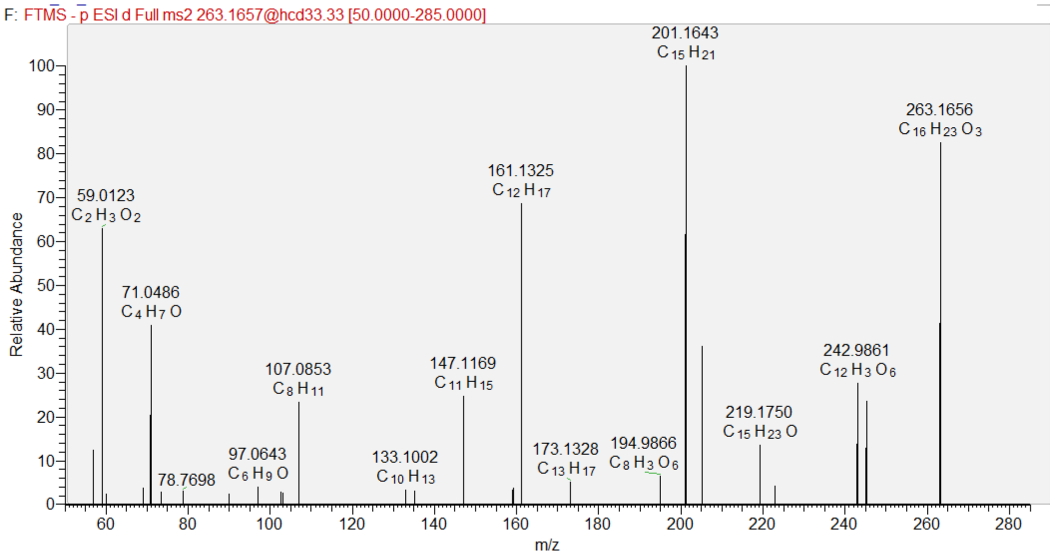

Figure S8. (-) ESI-MS/MS spectrum of hydroxyhexadecatetraenoic acid (**6**).
